# Nodular elastosis in the setting of lichen sclerosus

**DOI:** 10.1016/j.jdcr.2023.09.003

**Published:** 2023-09-24

**Authors:** Avni Patel, Marsha Chaffins

**Affiliations:** a Department of Dermatology, Henry Ford Hospital, Detroit, Michigan; b Department of Pathology, Henry Ford Hospital, Detroit, Michigan

**Keywords:** elastosis, lichen sclerosus, vulvar dermatology

## Introduction

Up to 20% of women experience vulvar discomfort during their lifetime.^1^ While there are many etiologies for this discomfort, lichen sclerosus (LS) is a common disfiguring condition seen by dermatologists. Typically accompanied by burning, itching, and pain, LS eventually progresses to complete resorption of normal genitalia and possible malignancy if not treated. While the classic appearance of LS is white to erythematous atrophic appearing patches, often with purpura and superficial erosions, variations in and lack of familiarity with anatomy may complicate a physician’s clinical recognition. Herein, we report a relatively novel clinical and histopathologic finding, which we suspect may be under-recognized: nodular elastosis in the setting of LS.

## Case reports

**Fig 1:**
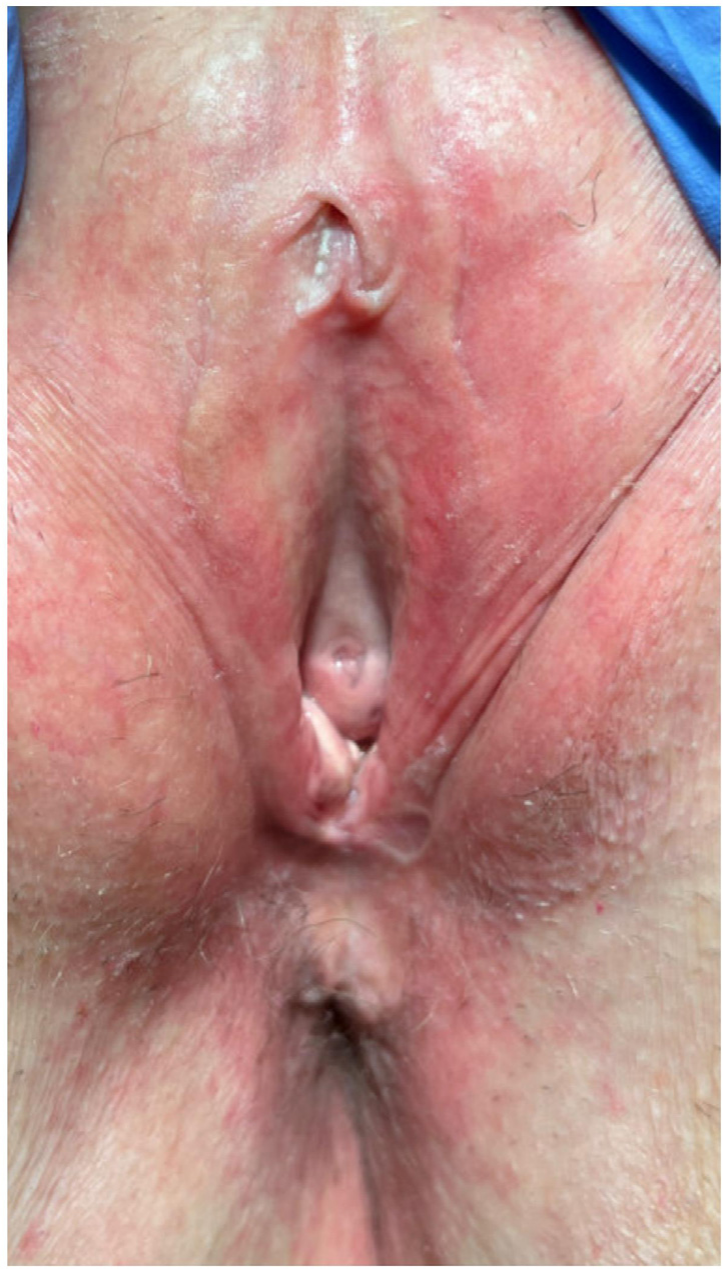
Erythema in a figure-of-8 pattern with complete agglutination of the labia minora and partial agglutination of the clitoral hood. Yellow nodules are visualized at the posterior introitus.

Case 1: A 53-year-old female presented to clinic with significant global vulvar pain and discomfort ongoing for 2 years. She endorsed chronic constipation and had previously undergone perineal biopsy consistent with LS. Physical examination demonstrated erythema in a figure-of-8 pattern with complete agglutination of the labia minora and partial agglutination of the clitoral hood (Fig 1). Incidentally, many firm, somewhat rubbery, yellow-to-white nodules and plaques were noted at the posterior introitus. The patient was unaware of their presence. The lesion was biopsied and revealed histologic changes characteristic of LS as well a broad zone of elastosis. Verhoeff van Geison stain highlighted masses of elastic fibers.

**Fig 2:**
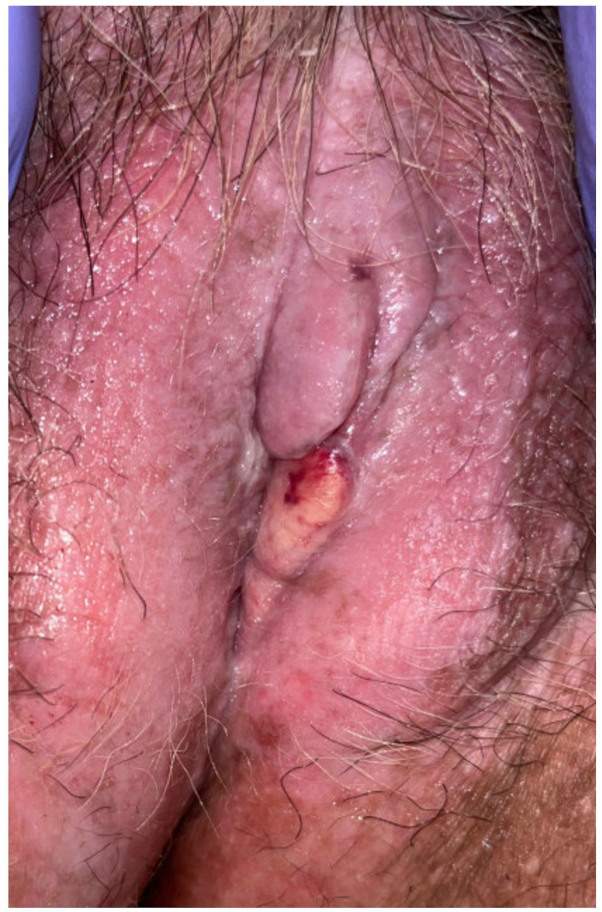
Firm, rubbery, nontender, yellow plaque with focal overlying hemorrhage on the left labium majus surrounded by pink, atrophic-appearing confluent patches. Note also complete agglutination of the bilateral labia minora and clitoral hood.

**Fig 3:**
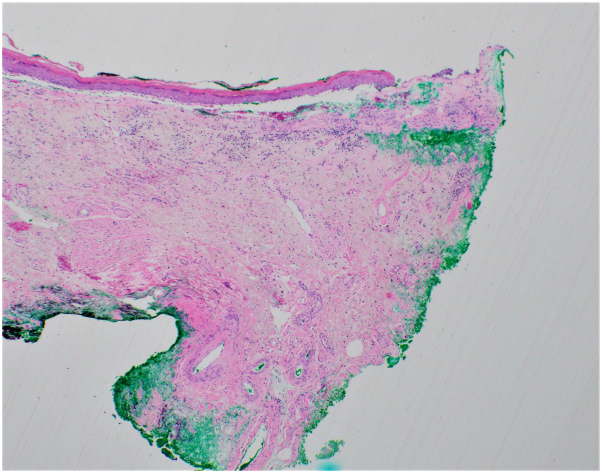
Edema and homogenization of papillary dermal collagen with secondary bullae formation.

**Fig 4:**
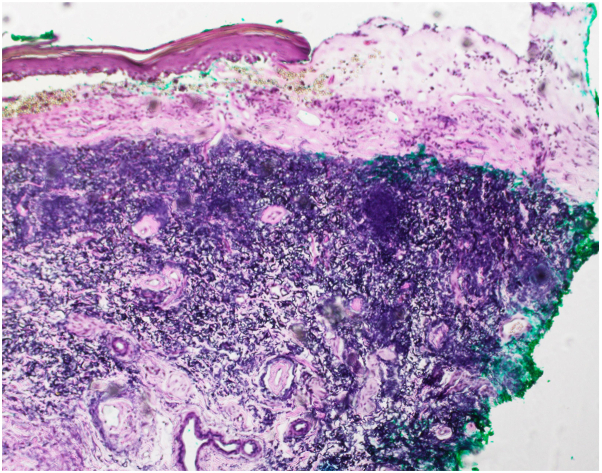
VVG stain revealed diminished elastic fibers in the superficial dermis with accumulation of elastic fibers below this zone. *VVG*, Verhoeff van Geison.

Case 2: A 70-year-old female presented to clinic with a 15-year history of global vulvar pain and pruritus accompanied by sores and fissures. She previously underwent biopsy of the right perineum, which favored LS. Physical examination demonstrated shiny, pink, atrophic-appearing patches of the bilateral labia majora and complete agglutination of the bilateral labia minora and clitoral hood. The left medial labium majus was incidentally noted to have a large, firm, rubbery, nontender, yellow plaque with focal overlying hemorrhage (Fig 2). Biopsy of this plaque demonstrated edema and homogenization of papillary dermal collagen with secondary bullae formation (Fig 3). In addition, Verhoeff van Geison stain revealed diminished elastic fibers in the superficial dermis with accumulation of elastic fibers below this zone (Fig 4).

## Discussion

While there are many studies in the literature about LS, there appears to be a paucity regarding the clinical appearance and development of vulvar elastosis in the setting of chronic inflammatory conditions. In 1999, Allen et al reported haphazardly arranged bundles of elastic fibers in the reticular dermis of a scar on the abdomen as “histologic findings of late-stage LS.”^2^ However, in recent years there has been growing histopathologic interest in this finding. Shiba et al studied biopsy samples from women with LS and found moderate to advanced levels of elastosis in 72% of vulvar samples.^3^ As in one of our cases, they noted a decrease of elastic fibers in the papillary dermis and an increase in the mid to deep dermis. Shiba et al hypothesized that this histologic pattern represents a repair process associated with this disease, and that this finding may be pathogenically important in scar formation.^3^

See et al^1^ conducted a larger histopathologic study to characterize vulvar elastosis as a novel entity. They found that 45% percent of all vulvar biopsies reviewed demonstrated some degree of vulvar elastosis and, interestingly, 68% of biopsies from women with LS demonstrated elastosis.^1^ Similar histologic features are seen in other elastotic entities, all of which are sun exposed (pseudoxanthoma elasticum, solar elastosis, etc.).^1^ It is unclear why these features are seen in a sun protected area such as the vulva. See et al hypothesized that the pathogenesis of vulvar nodular elastosis may occur as a degenerative phenomenon related to advanced age or hormone changes, as it has been noted in both inflammatory and noninflammatory conditions.^1^ There have also been reports of similar lesions in penile LS, previously termed “nevus elasticus.”^4^ We posit that nodular elastosis is a clinically under-recognized entity, and thus believe that clinical recognition and further study regarding pathogenesis and possible contribution to genital discomfort are warranted.

## Conflicts of interest

None disclosed.
